# Joint preservation surgery for correcting adolescents’ spasmodic flatfoot deformity: early results from a specialized North African foot and ankle unit

**DOI:** 10.1007/s00264-023-06011-5

**Published:** 2023-10-20

**Authors:** Amr A. Fadle, Ahmed A. Khalifa, Ahmed Bahy, Yousif T. El-Gammal, Hossam Abubeih, Wael El-Adly, Ahmed E. Osman

**Affiliations:** 1 Orthopedic Department, Assiut University Hospital, Assiut, Egypt; 2 Orthopedic Department, Qena Faculty of Medicine, South Valley University, Qena, Egypt

**Keywords:** Flatfoot, Pes planus, Spasmodic, Adolescents, Joint preservation

## Abstract

**Purpose:**

We aimed to report early results of performing joint-preserving surgeries for managing spasmodic flatfoot deformity (SFFD) in adolescents.

**Methods:**

A prospective case series study including 24 patients (27 feet) diagnosed with idiopathic SFFD not responding to conservative management. After reassessment under anesthesia, surgical procedures included soft tissue releases (Achilles tendon (AT), peroneus brevis (PB), peroneus tertius (PT) (if present), and extensor digitorum longus (EDL)), bony osteotomies (lateral column lengthening (LCL), medial displacement calcaneal osteotomy (MDCO), and double calcaneal osteotomy (DCO)), and medial soft tissue reconstruction or augmentation if needed. Functional evaluation was performed per the American Orthopedic Foot and Ankle Society (AOFAS) score, while radiological parameters included talo-navicular coverage angle (TNCA), talo-first metatarsal angle (AP Meary’s angle), calcaneal inclination angle (CIA), talo-calcaneal angle (TCA), talo-first metatarsal angle (Lat. Meary’s angle), and tibio-calcaneal angle (TibCA). The preoperative parameters were compared to the last follow-up using the Wilcoxon signed test.

**Results:**

The mean age was 15.37 ± 3.4 years, 18 (75%) were boys, and the mean BMI was 28.52 ± 3.5 (kg/m^2^). Release of AT and fractional lengthening of PL, PT, and EDL were performed in all patients. LCL was needed in eight feet (29.6%), MDCO in 5 (18.5%), and DCO in 14 (51.9%). FDL transfer was required in 12 (44.4%) feet, and repair of the spring ligament in seven (25.9%). The mean operative time was 99.09 ± 15.67 min. All osteotomies were united after a mean of 2.3 ± 0.5 months. After a mean follow-up of 24.12 ± 8.88 months (12 and 36 months), the AOFAS improved from a preoperative mean of 43.89 ± 11.49 to a mean of 87.26 ± 9.92 (*P* < 0.001). All radiological parameters showed significant improvement, AP Meary’s angle from a mean of 20.4 ± 5.3 to a mean of 9.2 ± 2.1, Lat. Meary’s angle from − 15.67° ± 6.31 to − 5.63° ± 5.03, TNCA from − 26.48° ± 5.94 to 13.63° ± 4.36, CIA from 12.04° ± 2.63 to 16.11° ± 3.71, TibCA from − 14.04° ± 3.15 to − 9.37° ± 3.34, and TCA Lat. from 42.65° ± 10.68 to 25.60° ± 5.69 (*P* ≤ 0.001). One developed wound dehiscence (over an MDCO), managed with daily dressings and local antibiotics. Another one developed lateral foot pain after having LCL managed by metal removal.

**Conclusion:**

Careful clinical and radiological evaluation for the correct diagnosis of SFFD is paramount. Joint-preserving bony osteotomies combined with selective soft tissue procedures resulted in acceptable functional and radiological outcomes in this young age group.

## Introduction

Flat foot deformity (FFD) or pes planovalgus (PPV) in children and adolescents, which is generally described as a loss of medial longitudinal arch with an excessive hindfoot valgus alignment and forefoot abduction [1, 2], is considered one of the most frequent complaints presented to orthopedic outpatient clinics; however, it is yet considered a poorly understood condition caused by various etiologies and no clear consensus regarding definitive lines of management [3–5].

This deformity could be broadly classified into two major categories according to the mobility of the subtalar joint into a flexible flat foot deformity (FFFD) and a rigid flat foot deformity (RFFD) (when a significant subtalar joint motion restriction is present); both could be idiopathic (primary) or secondary (neurological, traumatic, dystrophic, and other conditions) [2, 4, 6]. Defining the underlying cause of FFD is paramount for initiating a management plan, which usually starts with a conservative line and progresses to surgical intervention if the non-surgical modalities fail to improve the patient’s symptoms [3, 5, 7, 8].

Furthermore, a separate entity of RFFD is the spasmodic or spastic flatfoot deformity (SFFD), which Sir Robert Jones first described in 1897 [9], and a tarsal coalition was considered the commonest cause for such deformity; however, other causes could lead to SFFD, such as inflammatory conditions causing a secondary protective peroneal or peroneo-extensor muscles spasm, and anterolateral facet impingement [10–14]. However, if no cause could be detected, some authors called the condition an idiopathic SFFD [6, 15, 16].

Conservative lines for managing SFFD were reported in the literature [17, 18]; even more, surgical interventions, including subtalar and triple arthrodesis, were described [19, 20]. However, joint-preserving surgeries (bony osteotomies with or without soft tissue procedures) were rarely described for managing SFFD in adolescents. These offer the advantage of efficient deformity correction and achieving acceptable outcomes without disturbing foot growth and development in younger age groups [2, 21]. So, we aimed to report early results of performing joint-preserving surgeries for managing idiopathic SFFD in adolescents not responding to conservative management. Furthermore, to offer an algorithmic approach to management.

## Methods

After obtaining approval from our local ethical committee (IRB no. 17100629), we conducted a prospective case series study in a specialized foot and ankle unit at an Egyptian (North African) tertiary university hospital between May 2017 and May 2021.

We included adolescent patients who presented to our outpatient clinic with a rigid, painful flatfoot deformity of unclear etiology (confirmed by the clinical and radiological evaluation) and did not respond to conservative treatment (medical and physiotherapy) for at least six months. We excluded patients with rigid flatfoot deformity secondary to tarsal coalition, ankle or subtalar joints arthritis, previous foot fractures, inflammatory arthritis, neurological deficit, and tumours; those with severe trophic skin disorders; and those with general contraindications to any surgery such as poor circulation, concurrent infection, and other comorbidities. This resulted in including 24 patients (27 feet: three bilateral and 21 unilateral) during the study period.

### Patients’ evaluation

Besides collecting patients’ basic demographic characteristics, a detailed history was obtained regarding general health, recent weight gain, recent involvement in strenuous activities, recent or repetitive foot or ankle trauma, mode of onset, and duration of symptoms. In this series, we noticed that most patients complained of increased symptoms after standing for longer durations.

(A) General musculoskeletal examination: mainly to exclude causes of secondary SFFD, such as possible congenital anomalies and neurologic deficit. Furthermore, both hips and knees were examined to exclude other deformities.
(B) Local foot and ankle clinical examination and functional evaluation [1, 5, 22]: this was performed during patients’ visits to the outpatient clinic and the morning rounds after admission to the hospital. We evaluated the gait, foot posture, foot arch state during weight-bearing and non-weight-bearing, the site of tenderness, and the range of motion (ROM) of the ankle and subtalar joints (STJ). One crucial point that was noted is that although patients presented with rigid deformity during their initial assessment in the outpatient clinic, however, this deformity was less when the evaluation was performed during the early morning rounds, which supported the diagnosis of having SFFD following a spasm triggered by exhaustion due to standing for long periods. The American Orthopedic Foot and Ankle Society (AOFAS) Ankle–Hindfoot scale was used for detailed functional assessment preoperatively and during follow-up visits.
(C) Radiographic evaluation: standing anteroposterior (AP), lateral, and axial plain radiographs and foot computed tomography (CT) were performed [8, 23]: (1) In the AP view: talo-navicular coverage angle (TNCA) and talo-first metatarsal angle (AP Meary’s angle); (2) in the lateral view: calcaneal inclination angle (CIA), talo-calcaneal angle (TCA), and talo-first metatarsal angle (Lat. Meary’s angle); (3) in the axial view: the tibio-calcaneal angle (TibCA) was measured; (4) CT scan was performed preoperatively for all patients to exclude the presence of the tarsal coalition bar and any joint arthritic changes.

### Operative details

**Fig. 1:**
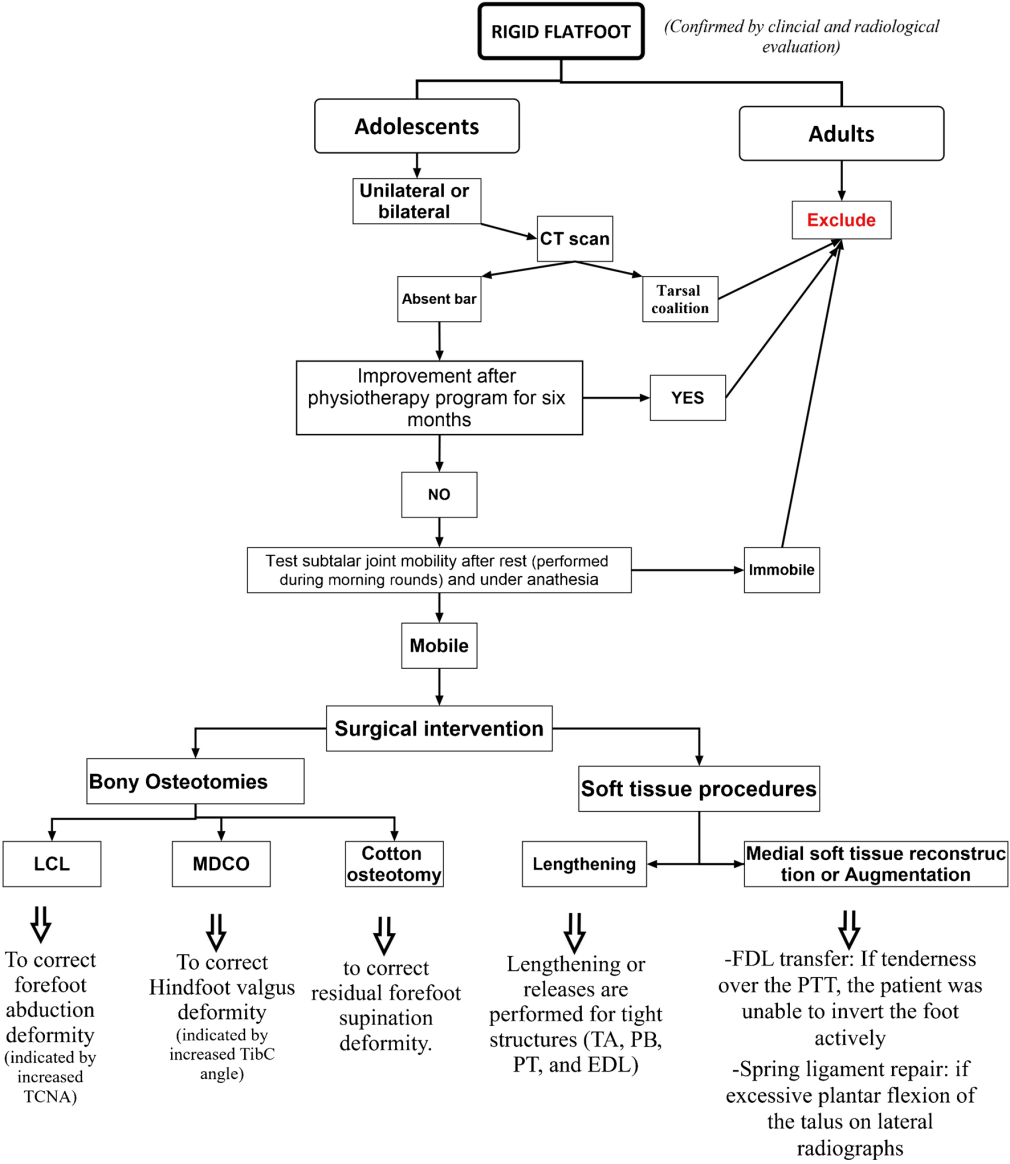
Patient selection and management algorithm

The surgical procedures were decided according to an algorithm demonstrated in Fig. 1.

All surgeries were performed under spinal anaesthesia while the patients were supine on a radiolucent operative table, with a support under the ipsilateral buttock to aid access to the lateral side of the foot, and a tourniquet was applied in all cases. Fluoroscopy was available in all surgeries. The first step was to examine the foot and ankle under anaesthesia to assess deformity correctability and recheck the ankle and STJ range of motion and notice the degrees of its recovery in the absence of pain. A combination of the following procedures (bony osteotomies and soft tissue interventions) was performed according to the previously mentioned algorithm; however, some changes could be performed according to the under-anaesthesia evaluation and the intraoperative findings.

(A) Soft tissue release(s): these were performed according to tight structures and the residual loss of ROM after evaluation under anaesthesia; Achilles tendon (AT) lengthening, if needed, was performed as the initial step through a Strayer technique for gastrocnemius recession. Then, various soft tissue fractional lengthening was performed as needed for the following structures: peroneus brevis (PB), peroneus tertius (PT) (if it is present), and extensor digitorum longus (EDL).
(B) Bony procedures: (1) medial displacement calcaneal osteotomy (MDCO): a transverse osteotomy was performed through a lateral incision; fixation was achieved by two screws (either cannulated 7.3-mm or 6.5-mm cannulated headless screws) in older patients; however, in younger patients, we used smooth pins. (2) Lateral column lengthening (LCL): this was performed according to Mosca modification [24]; an Iliac bone graft or mini wedge plate was used to keep the amount of distraction. (3) Combined osteotomy (double calcaneal osteotomy (DCO)): meaning that an LCL and MDCO were performed on the same foot. It was indicated if there was a combined increased hindfoot valgus with forefoot abduction (as noted by increased TNCA). Particular attention should be paid to planning skin incisions to avoid skin and wound healing complications. Another important consideration is the direction of the screws used to fix the MDCO; they were placed in a way that does not interfere with the lateral plate placement used for the fixation of the LCL osteotomy. (4) Additional procedures: (a) cotton osteotomy (medial cuneiform osteotomy): which was performed to correct residual forefoot supination deformity (either it could be a dorsal opening wedge osteotomy (as described originally) or it could be performed as plantarly closing wedge osteotomy which will assist in reducing the medial longitudinal arch length, if present [25]), and (b) navicular-cuneiform fusion, which was performed to restore the arch if there was a midfoot collapse.
(C) Medial soft tissue repair or augmentation: in patients with dysfunction of the posterior tibial tendon (PTT) (suspected during clinical evaluation by local tenderness over the tendon and inability to invert the foot actively) and in cases with spring ligament tear (indicated by excessive plantar flexion of the talus on lateral radiographs), a medial soft tissue reconstructive procedure was performed through a medial incision, either by flexor digitorum longus (FDL) transfer to augment the PTT or by anatomical repair or plication of the spring ligament.

### Postoperative and follow-up protocols

(A) During hospital stay: immediate postoperatively, patients were placed in a well-padded posterior slab, and the neurovascular status of the foot was observed. Initial postoperative plain radiographs were used for assessing the correction and implant positioning. The physiotherapy program started on the first postoperative day, mainly on hip abductors and quadricep muscle strengthening. Patients were usually discharged on postoperative day two unless severe swelling or pain was encountered, which was closely monitored till it was relieved.
(B) Follow-up: the first follow-up visits were scheduled after two weeks, where wounds were checked, sutures were removed, and the slab was changed to a short leg cast (for a further 4 weeks), kept in slight inversion if medial soft tissue reconstruction was performed. The second visit was after six weeks, where the cast was removed and replaced by a brace, and a radiographic evaluation was performed using the same preoperative radiographic series (to assess alignment and bony union). If radiographs showed a good union of osteotomies, a programmed physiotherapy protocol aiming for ankle and STJ mobilization, starting with inversion and plantar flexion, was started, then gradually increasing the range to restore the ankle’s dorsiflexion and the STJ eversion. Stretching of the foot evertors and toe dorsiflexors, as well as strengthening of the tibialis posterior muscle, was encouraged throughout the program.

**Fig. 2:**
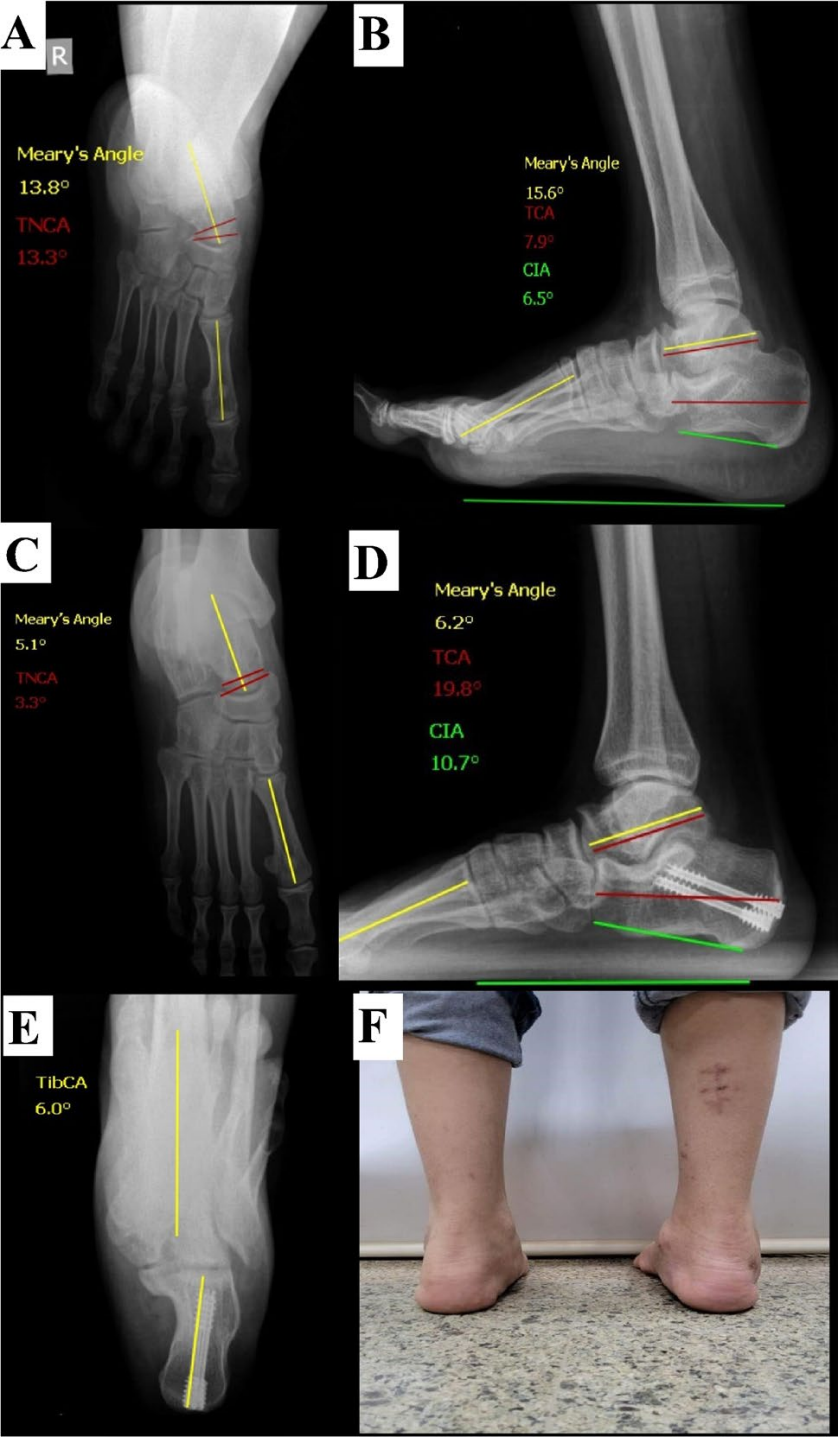
A male patient, 14 years old, had an MDCO procedure. **A**, **B** Preoperative radiographs. **C**–**E** Last follow-up (18 months) radiographs showing deformity correction (as per angles measured) and restoration of the heel alignment as shown in the axial view. **F** A clinical image showing restoration of the heel alignment

**Fig. 3:**
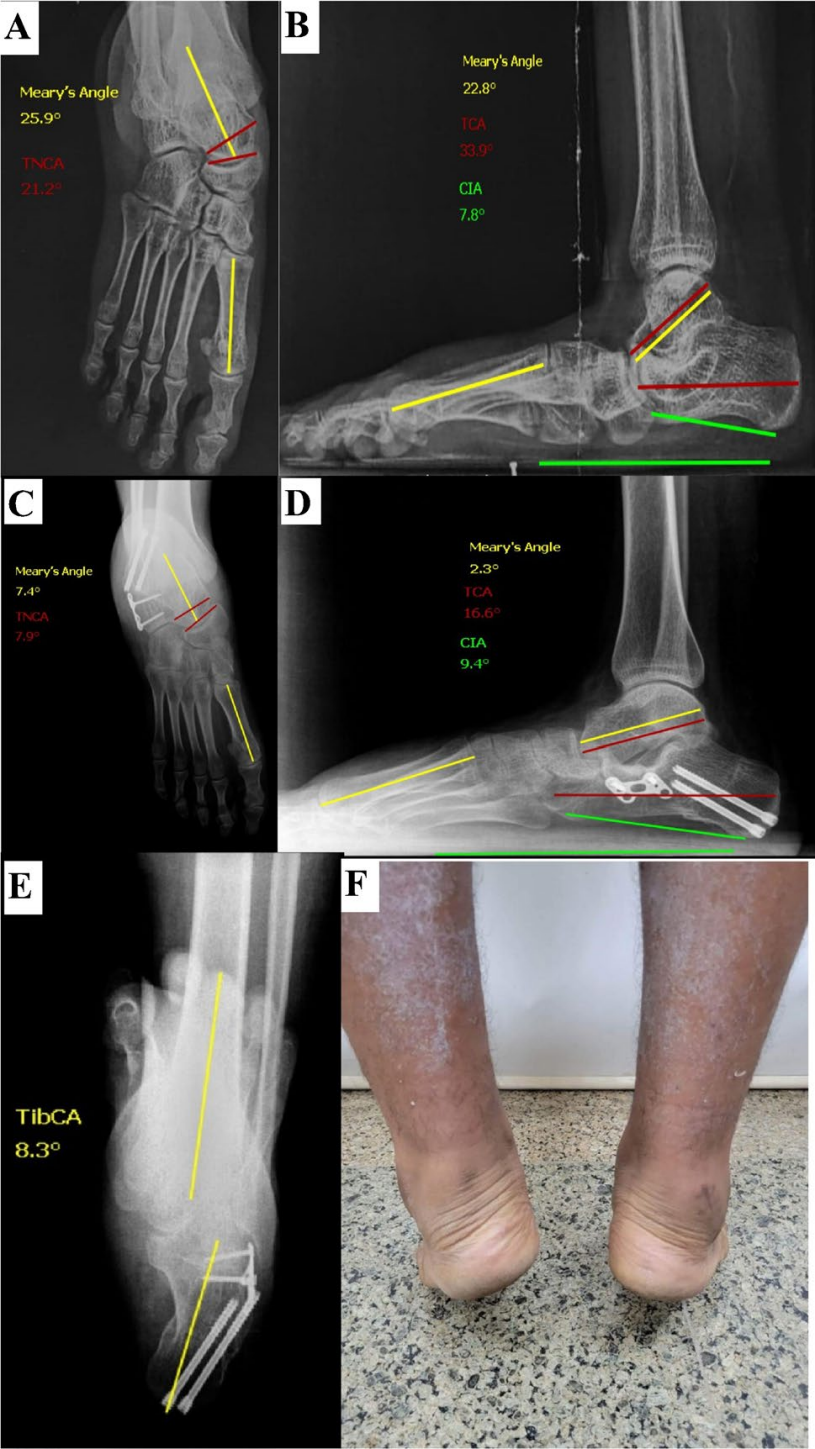
A male patient, 17 years old, had a DCO procedure. **A**, **B** Preoperative radiographs. **C**–**E** Last follow-up (20 months) radiographs showing deformity correction (as per angles measured) and restoration of the heel alignment as shown in the axial view. **F** A clinical image showing restoration of the heel alignment

**Fig. 4:**
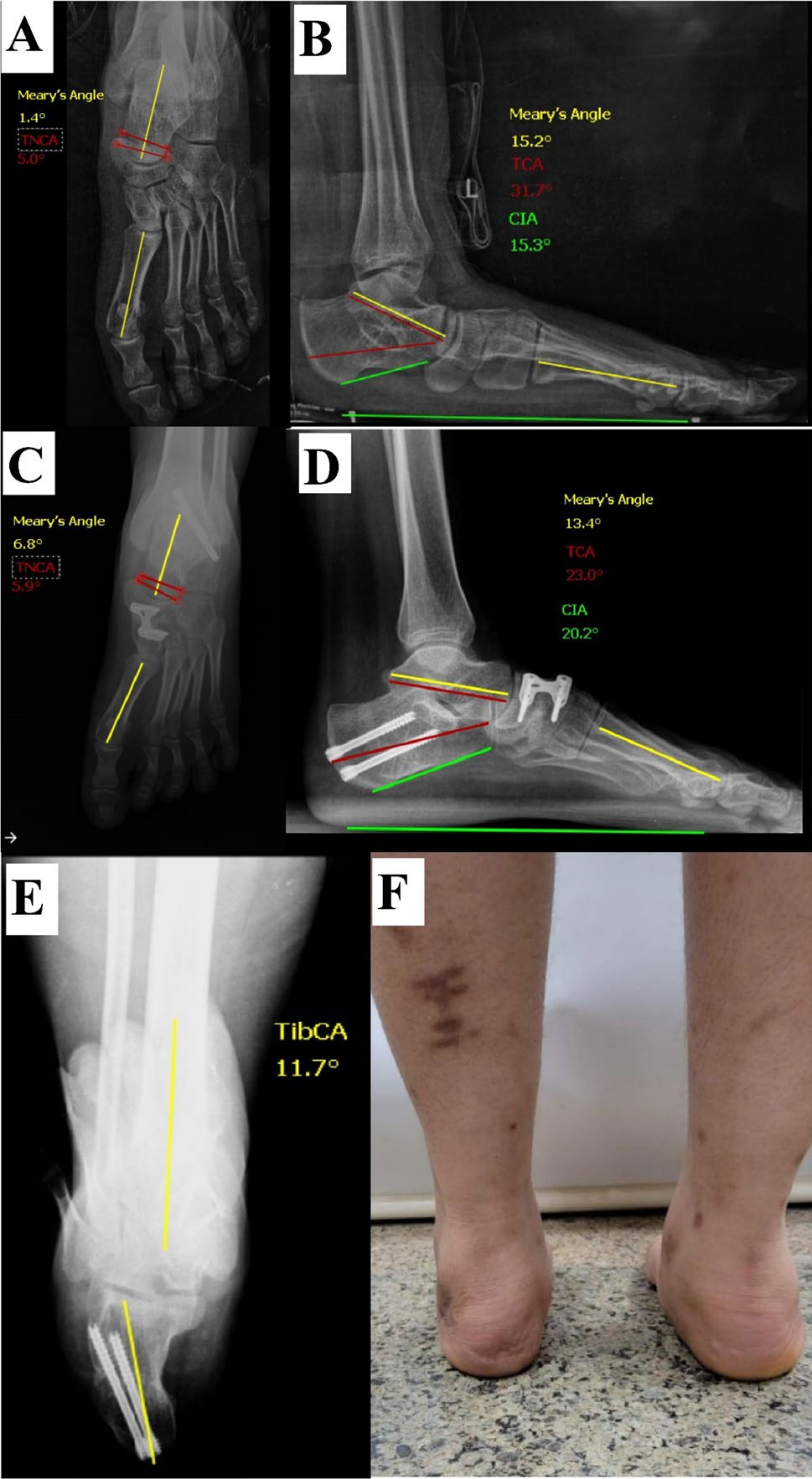
A female patient, 15 years old, had an MDCO combined with a naviculo-cuneiform fusion. **A**, **B** Preoperative radiographs. **C**–**E** Last follow-up (16 months) radiographs showing deformity correction (as per angles measured) and restoration of the heel alignment as shown in the axial view. **F** A clinical image showing restoration of the heel alignment

Then, follow-up visits were scheduled at three, six, and 12 months and then annually. Functional and radiological assessments were performed, and the last follow-up data were reported and compared to the preoperative evaluation data (Figs. 2, 3, and 4). Any complications during the course of management or follow-up were documented and reported. All patients were available during the last follow-up.

### Statistical analysis

Data was analyzed using the Statistical Package for Social Sciences (SPSS), version 26.0 for Windows. Qualitative data is expressed as frequency and percent, and quantitative data is tested for normality by the Shapiro–Wilk test and expressed as mean ± SD and 95% CI (confidence interval). The Wilcoxon signed test was used to compare the mean difference between preoperative and postoperative follow-up data. The level of significance was considered at a *P* value < 0.05.

## Results

(A) Basic patients’ characteristics: the final analysis included 24 patients (27 feet), the details mentioned in Table 1. The mean duration of complaint was 11.74 ± 4.1 months (range from 6 to 24). Patients were followed up for a mean of 24.12 ± 8.88 months (12 and 36 months). All osteotomies were united after a mean of 2.3 ± 0.5 months (range from 2 to 4.5).
(B) Operative data: the mean operative time was 99.09 ± 15.67 min (80 and 125 min). Release of AT was performed as an initial step in all patients; furthermore, fractional lengthening of PL, PT, and EDL was performed in all patients. Bony procedures needed were as follows: LCL in eight feet (29.6%), MDCO in 5 (18.5%), DCO in 14 (51.9%), cotton osteotomy in two (7.4%), and naviculo-cuneiform fusion in two (7.4%) (Table 1). FDL transfer was required in 12 (44.4%) feet, while the repair of the spring ligament was required in seven (25.9%).
(C) Functional and radiological outcomes: the AOFAS score improved from a preoperative mean of 43.89 ± 11.49 to a postoperative mean of 87.26 ± 9.92 (*P* < 0.001). Ankle joint and STJ motion improved compared to preoperative measurements, and all radiological parameters showed significant improvement during the last follow-up, as shown in Table 2.
(D) Complications: all patients had no recorded postoperative complications except for two patients. One developed wound dehiscence (over an MDCO), managed with daily dressings and local antibiotics. The other developed lateral foot pain after having LCL, which continued postoperatively for over a year and was managed by metal removal. There was no reported delayed union or non-union of the osteotomies in any patient.

**Table 1:** Basic demographic and operative details

Variables	
Patients’ characteristics	
No. of patients	24
Age^†^	15.37 ± 3.4 years (from 10 to 18)
Gender^‡^	18 (75%) boys Six (25%) girls
No. of feet	27 (3 bilateral, 21 unilateral)
Laterality^‡^	14 (51.9%) left 13 (48.1%) right
BMI	The overall mean was 28.52 ± 3.5 (kg/m^2^)^†^: -Six (25%) patients have normal weight^‡^ -Ten (41.7%) patients were overweight^‡^ -Eight (33.3%) patients were obese^‡^
Bony osteotomies operative details^‡^	
DCO	14 feet (51.9%)
LCL	Only: in eight feet (29.6%)
	Total: in 22 feet (81.5%)
MDCO	Only: in five feet (18.5%)
	Total: in 19 feet (70.4%)
Cotton osteotomy	Two feet (7.4%)
Naviculo-cuneiform fusion	Two feet (7.4%)

*No.* number, *BMI* body mass index, *DCO* double calcaneal osteotomy, *LCL* lateral column lengthening, *MDCO* medial displacement calcaneal osteotomy

^†^Data presented as mean SD (range)

^‡^Data presented as number (percentage)

**Table 2:** The clinical and radiological outcomes for the study group

Variables	Preoperative^†^	Last follow-up†	*P* value^‡^
Clinical assessment			
AOFAS	43.89 ± 11.49 (39.34–48.44)	87.26 ± 9.92 (83.33–91.19)	< 0.001
Range of motion			
Ankle dorsiflexion	27.74 ± 5.64 (25.74–28.26)	26.84 ± 6.28 (24.42–27.24)	< 0.001
Ankle plantar flexion	12.11 ± 12.39 (10.23–13.78)	29.74 ± 11.36 (27.63–30.54)	< 0.001
STJ inversion	7.21 ± 3.24 (5.36–8.23)	16.84 ± 7.67 (15.69–18.21)	< 0.001
STJ eversion	0.26 ± 1.15 (0.11–0.32)	8.94 ± 5.16 (7.54–9.25)	< 0.001
Radiological angles (°)			
AP Meary’s angle	20.4 ± 5.3 (18.56–21.57)	9.2 ± 2.1 (7.89–10.56)	< 0.001
TNCA	26.48 ± 5.94 (24.13–28.83)	13.63 ± 4.36 (11.90–15.36)	< 0.001
Lat. Meary’s angle	15.67 ± 6.31 (13.17–18.16)	5.63 ± 5.03 (4.19–7.81)	< 0.001
TCA Lat	42.65° ± 10.68 (40.36–43.25)	25.60° ± 5.69 (24.13–26.84)	< 0.001
CIA	12.04 ± 2.63 (10.99–13.08)	16.11 ± 3.71 (14.64–17.58)	< 0.001
TibCA	14.04 ± 3.15 (12.79–15.29)	9.37 ± 3.34 (8.05–10.69)	< 0.001

*TNCA* talo-navicular coverage angle, *CIA* calcaneal inclination angle, *TCA* alo-calcaneal angle, *TibCA* tibio-calcaneal angle

^†^Data are expressed as mean ± SD (95% confidence interval)

^‡^Wilcoxon signed test compares the mean difference between preoperative and postoperative last follow-up data

## Discussion

The main finding of the current study is that joint-preserving surgeries, including mainly bony osteotomies (either LCL or MDCO or DCO) in association with selected soft tissue procedures, are a promising option for managing SFFD when conservative lines fail, leading to acceptable functional and radiological outcomes with a high safety margin, especially in younger patients where foot mobility needs to be preserved for better functionality. The selection of which procedure to perform depends on preoperative clinical and radiological evaluation and intraoperative findings and could be tailored for each patient according to the severity of deformity and tight or deficient soft tissue structures, as summarized in Fig. 1.

Spasmodic or spastic flatfoot or sometimes called “neurological” is a term that describes a phenomenon caused by various patterns of muscle spasms (peroneal muscle (mainly the peroneus brevis) and extensor digitorum longus) leading eventually to this specific subtype of RFFD [6, 8].

The mechanism by which SFFD develops could be explained by the presence of free nerve endings around the sinus tarsi, which are responsible for receiving nociceptive stimuli, motor sensation, and joint positioning. Stimulation of these nerve endings by chronic subtalar joint irritation triggered by many factors, including and not limited to synovitis [18], post-traumatic [26], and accessory anterolateral talar facet [12], will lead to spasticity of the peroneal muscle group through stimulation of reflex arcs and development of SFFD as a protective response to splint the subtalar and ankle joints to alleviate pain [18, 27]. Furthermore, this splinting mechanism usually results in functional shortening or contracture of the musculotendinous unit (peroneus brevis commonly, peroneus longus, and extensor digitorum longus) [18, 28].

It is widely accepted that the peroneal or peroneo-extensor muscle complexes are not always spasmodic; however, these structures could be shortened (an organic shortening rather than a neurologic clonus) to adapt to the chronically everted hindfoot with a concomitant limited subtalar motion [11, 18, 28].

Furthermore, this condition should be differentiated from SFFD secondary to other conditions causing muscle contractures or chronic spasms, such as cerebral palsy (particularly in patients with diplegia and quadriplegia) and arthrogryposis multiplex congenita [29, 30]. However, it could be called idiopathic if no specific cause could be detected [6, 15, 16].

In the current study, we excluded any other causes leading to SFFD by detailed history and clinical and radiological evaluation; furthermore, to ensure the absence of tarsal coalition, all patients had CT evaluation. A vital remark regarding patients included in our study is that most patients reported standing for long periods, 75% were either obese or overweight, and some reported a history of ankle or foot trauma, but the details were not precise.

The role of involvement in strenuous activities, being overweight, and exposing the foot and ankle to trauma in triggering SFFD was confirmed in previous studies. Todd, in 1939 [31], reported that 5.7% of individuals with FFD had the unique entity of SFFD; he reported that those were adolescent patients who were involved in laborious work requiring standing for long periods; furthermore, he mentioned that in most of the unilateral cases, a history of foot and ankle trauma was detected, and in bilateral cases, the severity was not equal. Rizk and Kandil [17] and Blockey [13] reported that their patients started complaining at 12 to 14 years old after being involved in strenuous activities or having repeated traumas. Furthermore, Pauk and Ezerskiy cautioned about being overweight combined with an increase in physical activities as risk factors for FFD complication progression [32].

Regardless of SFFD aetiology, a management plan should be initiated if this condition becomes symptomatic, starting with conservative lines such as physiotherapy, serial casting, and local injections, up to surgical correction in refractory cases [17, 18].

Conservative management, including local sinus tarsi injection, serial casting, stretching exercises, insoles, common peroneal nerve block, and abstaining from sports activities, proved efficacy in some reports [12, 17, 18, 26]. However, the caveats of these lines are that it mainly depends on the patient’s compliance to the instructions, orthoses might worsen the condition as trying to invert the rigidly everted subtalar joint or dorsiflex a rigidly plantarflexed ankle joint will eventually exacerbate the pain, and the unpredicted rate or recurrence, reaching up to 40% of complete relapse [1, 13, 17]. In the current study, we exhausted conservative management lines (mainly medical and physiotherapy) for at least six months before deciding on surgical management.

In a study by Rizk and Kandil [17], including 50 feet with SFFD secondary to peroneal or peroneo-extensor spasm in 33 patients having a mean age of 14 + 2.8 years, they managed all their cases conservatively by manipulation under anaesthesia, local steroid injection to the sinus tarsi, and a walking cast. By a mean last follow-up of 22.5 ± 3.5 months, they reported significant improvement in the AOFAS score from 40.9 + 3.5 at presentation to 73.56 + 5.2 (*P* < 0.001); however, they reported that only 12 feet were painless and mobile, and partial or complete relapse occurred in 38 (76%) feet. Furthermore, a complete relapse with unsatisfactory outcomes was reported in 14 (28%) feet; five developed arthritic changes and were treated with triple arthrodesis.

The surgical management of SFFD varied among studies and was mainly performed to attack the original pathology, and rarely joint-preserving surgeries were reported [6, 19, 20, 33]. We managed all our patients using mainly bony osteotomies and supplementary soft tissue procedures based on a predetermined management protocol based on data obtained preoperatively during the patient evaluation phase (clinical and radiological) augmented by data obtained during surgery and after repeating evaluation under anaesthesia. Till the last follow-up, we obtained acceptable functional and radiological outcomes.

Performing hindfoot osteotomies with soft tissue procedures (releases and medial side reconstruction) will assist in maintaining the medial column stability and restoring the foot arch; the combination of these procedures resulted in acceptable outcomes, as Wen et al. reported [34]. MDCO is performed to correct severe hindfoot valgus; it translates the weight-bearing and medializes AT insertion, which protects the STJ and reduces lateral ankle impingement; in addition, it will abolish the AT deforming force, which improves FDL transfer outcomes [34, 35]. LCL procedure is mainly for correcting the forefoot abduction deformity (indicated by increased TNCA); however, in severe deformity, LCL alone will lead to an increase in the lateral plantar pressure with persistent pain to reduce this possible increased pressure; another procedure, such as cotton osteotomy could be added [36, 37]; Xu et al. showed acceptable results combining both osteotomies (DCO) for managing severe adolescent FFD [36]. It is to be noted that when performing DCO, planning skin incisions and proper selection of osteotomy sites and fixation devices are paramount for success.

In a study by Luhmann et al. [6], the authors reported that 13 feet (nine patients) having a mean age of 14 ± 6 years were diagnosed with SFFD; after conservative management failure, the authors managed these cases surgically; first, an examination under anaesthesia was performed to confirm the correctability of the deformity followed by local injection; however, if the peroneal tendon remained contracted with a limited subtalar joint motion, they performed a fractional lengthening of the peroneus brevis and longus; the authors reported applying a short leg cast in full inversion for all patients. Three (32.1%) feet had subsequent surgical intervention, one had calcaneal neck lengthening with further peroneal lengthening, and two had subtalar arthrodesis. Of note, the authors mentioned that all of their patients were considered overweight compared to normal individuals in their age.

Martus et al. first reported the presence of an SFFD in association with accessory anterolateral talar facet AALTF in adolescents, which further worsens with overactivities and increased weight [33]; besides resecting AALTF if present, Martus et al. described performing soft tissue procedures such as gastrocnemius recession and bony osteotomies such as LCL and MDCO [33].

Some authors advocated triple arthrodesis for managing SFFD (especially in patients with a neuromuscular disorder); although the foot could be realigned, this management option was associated with deformity under-correction, non-union, and decreased ROM, and the foot loses its shock-absorbing function, which eventually led to an increased risk of adjacent joint arthritis reaching up to 40% [5, 38, 39]. So, we preferred to manage the patients with joint-reserving options in association with selective soft tissue releases or augmentation, which we consider advantageous, and avoid complications associated with arthrodesis options, especially when dealing with younger populations.

The current study had some inherent limitations. First, the relatively small number of included patients. Second, this was a non-comparative study; no other modalities (such as injection and advanced physiotherapy programs) were compared. Third, the follow-up is relatively short, and a longer follow-up is needed to track the progression of those patients regarding deformity recurrence or development of arthritis. Last, although we proposed an algorithm for management, we admit that it is still immature owing to the heterogeneity of the performed procedures on a relatively small number of patients. Furthermore, clear indications for each procedure were difficult to determine due to possible intraoperative circumstances forcing the surgeon to add or remove a surgical step; we hope that in light of the newly introduced classification system [40] (which was published after we started our study), we would be able to formulate more clear indications for each procedure according to different deformity stages.

## Conclusion

Careful clinical and radiological evaluation for correctly detecting the possible cause of SFFD is paramount for successful management. Conservative management should be exhausted first before deciding on surgical interventions. Joint-preserving bony osteotomy combined with selective soft tissue procedures based on preoperative and intraoperative data resulted in acceptable functional and radiological outcomes in this young age group. However, longer follow-up and further well-designed comparative studies are needed to confirm the results obtained from the current study.

## Data Availability

All the data related to the study are mentioned within the manuscript; however, the raw data are available with the corresponding author and will be provided upon a written request.

## Declarations

### Study setting

Foot and Ankle Surgery Unit, Orthopaedic Department, Assiut University Hospital, Assiut, Egypt.
